# Supplementary material for: Hybrid Models and Biological Model Reduction with PyDSTool
Source: PLoS Comput Biol. 2012 Aug 9;8(8):e1002628. doi: 10.1371/journal.pcbi.1002628 (PMC3415397; doi:10.1371/journal.pcbi.1002628)
Supplement: Text S4 — Complete source code for the PyDSTool package (version 0.88.120504). Includes API documentation and help files linking to web pages. This file is identical to the current public release on Sourceforge.net. (ZIP) [file pcbi.1002628.s004.zip › PyDSTool/html/PyDSTool.Toolbox.optimizers.optimizer-module.html]

xml version="1.0" encoding="ascii"?


PyDSTool.Toolbox.optimizers.optimizer


| Home | Trees | Indices | Help | PyDSTool |
| --- | --- | --- | --- | --- |

Package PyDSTool ::
Package Toolbox ::
Package optimizers ::
Package optimizer

[hide private]

[frames] | no frames]

# Package optimizer

source code

Module containing the core optimizers

Optimizers :

- Optimizer
  - a skeletton for defining a custom optimizer
  - calls iterate until the criterion indicates that the optimization
    has converged
- StandardOptimizer
  - takes a full step after a line search
- StandardOptimizerModifying
  - takes a full step after a line search
  - modifies the resulting parameters


|  |  |
| --- | --- |
| Submodules | [hide private] |

- **PyDSTool.Toolbox.optimizers.optimizer.optimizer**: *The core optimizer from which every other optimizer is derived*
- **PyDSTool.Toolbox.optimizers.optimizer.standard\_optimizer**: *A standard optimizer*
- **PyDSTool.Toolbox.optimizers.optimizer.standard\_optimizer\_modifying**: *A standard optimizer with a special object that modifies the
  resulting set of parameters*

  


|  |  |
| --- | --- |
| Classes | [hide private] |

StandardOptimizer  
A standard optimizer, takes a step and finds the best candidate
Must give in self.optimalPoint the optimal point after optimization

StandardOptimizerModifying  
A standard optimizer, takes a step and finds the best candidate
Must give in self.optimalPoint the optimal point after optimization
After each iteration the resulting optimization point is modified
by a call to a function


|  |  |
| --- | --- |
| Variables | [hide private] |

optimizer\_\_all\_\_ = `['StandardOptimizer', 'StandardOptimizerMod...`


|  |  |
| --- | --- |
| Variables Details | [hide private] |

### optimizer\_\_all\_\_

Value:
:   ```
    ['StandardOptimizer', 'StandardOptimizerModifying']
    ```

  


| Home | Trees | Indices | Help | PyDSTool |
| --- | --- | --- | --- | --- |

|  |  |
| --- | --- |
| Generated by Epydoc 3.0.1 on Fri May 4 15:24:05 2012 | http://epydoc.sourceforge.net |
